# Epigenetic patterns newly established after interspecific hybridization in natural populations of *Solanum*

**DOI:** 10.1002/ece3.758

**Published:** 2013-09-09

**Authors:** Nicolás Cara, Carlos F Marfil, Ricardo W Masuelli

**Affiliations:** 1 Facultad de Ciencias Agrarias, Instituto de Biología Agrícola Mendoza (IBAM), Universidad Nacional de CuyoA. Brown 500 (M5528AHB), Chacras de Coria, Mendoza, Argentina; 2 Instituto Nacional de Tecnología Agropecuaria (INTA), E. E. A. La ConsultaMendoza, Argentina

**Keywords:** Adaptation, DNA methylation, hybridization, wild potatoes

## Abstract

Interspecific hybridization is known for triggering genetic and epigenetic changes, such as modifications on DNA methylation patterns and impact on phenotypic plasticity and ecological adaptation. Wild potatoes (*Solanum*, section *Petota*) are adapted to multiple habitats along the Andes, and natural hybridizations have proven to be a common feature among species of this group. *Solanum* *× rechei*, a recently formed hybrid that grows sympatrically with the parental species *S. kurtzianum* and *S. microdontum*, represents an ideal model for studying the ecologically and evolutionary importance of hybridization in generating of epigenetic variability. Genetic and epigenetic variability and their correlation with morphological variation were investigated in wild and ex situ conserved populations of these three wild potato species using amplified fragment length polymorphism (AFLP) and methylation-sensitive amplified polymorphism (MSAP) techniques. We observed that novel methylation patterns doubled the number of novel genetic patterns in the hybrid and that the morphological variability measured on 30 characters had a higher correlation with the epigenetic than with the genetic variability. Statistical comparison of methylation levels suggested that the interspecific hybridization induces genome demethylation in the hybrids. A Bayesian analysis of the genetic data reveled the hybrid nature of *S*. × *rechei*, with genotypes displaying high levels of admixture with the parental species, while the epigenetic information assigned *S*. × *rechei* to its own cluster with low admixture. These findings suggested that after the hybridization event, a novel epigenetic pattern was rapidly established, which might influence the phenotypic plasticity and adaptation of the hybrid to new environments.

## Introduction

Hybridization and polyploidization are important evolutionary forces in plants, as a source of genetic and epigenetic variation. Interspecific hybridizations generate genetic alterations and modifications on DNA methylation (Salmon et al. 2005; Marfil et al. 2006). The latter is considered an epigenetic mark, along with histone modifications and regulation by small RNAs. Epigenetic variations generate new phenotypic variants that could be targeted by selection (Kalisz and Purugganan 2004; Rapp and Wendel 2005). Also, in many cases, epigenetic marks can be transmitted across generations operating as genuine genetic variation, and therefore, they could play a significant role in speciation and evolution (Jablonka and Raz 2009). Amplified fragment length polymorphism (AFLP) and methylation-sensitive amplification polymorphism (MSAP) analysis showed that hybridization more than polyploidization triggered epigenetic changes in the allopolyploid genomes of *Spartina* (Ainouche et al. 2004), *Senecio* (Hegarty et al. 2011), and *Arabidopsis* (Wang et al. 2006). In recent years, some studies assessing the importance of epigenetic variation in natural populations were published (Salmon et al. 2005; Marfil et al. 2009; Paun et al. 2010; Lira-Medeiros et al. 2010), but it is necessary to increase empirical evidence for natural epigenetic variation.

Wild potatoes (*Solanum*, section *Petota*) constitute an ideal model to analyze the evolutionary consequences of hybridization in the generation of epigenetic variation and its significance in the phenotypic plasticity and ecological adaptation: (i) Natural hybridization and hybrid speciation have been proposed to be prevalent in this group of plants (Hawkes 1990; Masuelli et al. 2009); (ii) wild potato species are adapted to multiple habitats along the Andes in America and represent a group with extensive phenotypic plasticity (Hawkes 1990); and (iii) both modes of reproduction in wild potatoes, sexual by seeds and asexual by stolons and tubers, would allow the study of transgenerational inheritance and mitotic stability of epialleles.

Analyzing synthetic hybrids between *Solanum tuberosum* and *Solanum kurtzianum,* we observed DNA methylation changes induced by hybridization (Marfil et al. 2006). In the natural hybrid *Solanum ruiz-lealii,* a relationship between methylation patterns and abnormal floral morphology was observed (Marfil et al. 2009). In this study, we expect to extend the observations described above by analyzing natural populations of an interspecific hybrid and its parental species.

**Figure 1:**
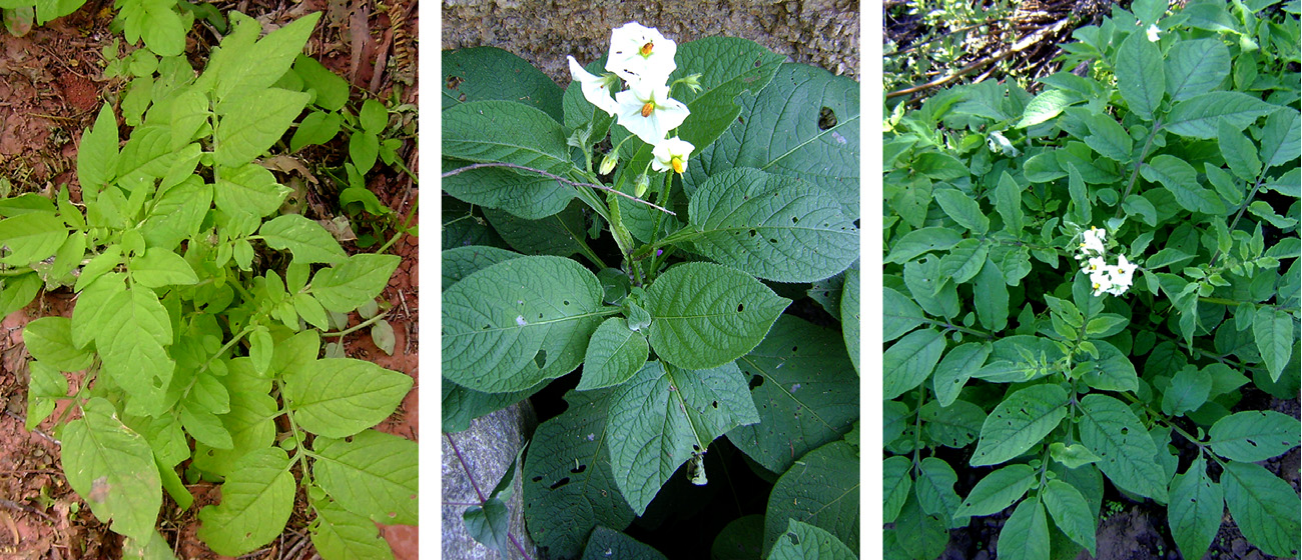
The three wild potato species studied growing in their natural habitats: the hybrid *Solanum* × *rechei* (right) and the parental species *Solanum kurtzianum* (left) and *Solanum microdontum* (center).

**Figure 2:**
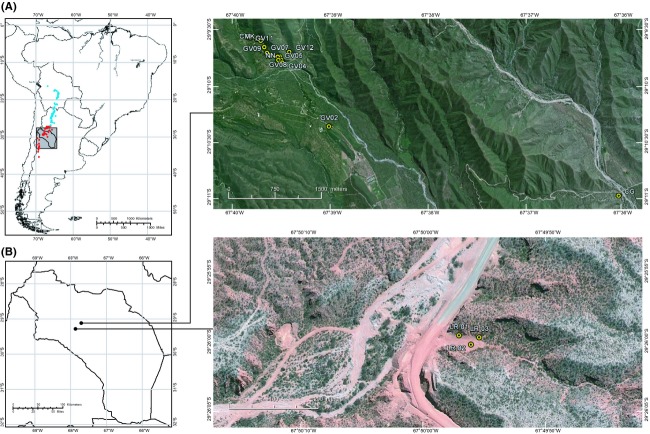
Distribution of *Solanum kurtzianum* and *Solanum microdontum* and sampled regions in this work. (A) Distribution of *S. kurtzianum* (red, Argentina) and *S. microdontum* (blue, Argentine and Bolivia). (B) Map of La Rioja province and detail of two regions where natural populations of *S. kurtzianum*, *S. microdontum*, and *Solanum* × *rechei* were collected (yellow).

Of 27 taxa of putative hybrid origin within section *Petota* (Spooner and van den Berg 1992), *Solanum* × *rechei* (Hawkes *et* Hjerting) is one of the most extensively documented (Fig. 1). The distribution of *S*. × *rechei* is restricted to the locality of Guanchín, at the southern end of the Sierra de Famatina in Argentina, province of La Rioja, department of Chilecito. In this locality, the parental species *S. kurtzianum* (Bitter *et* Wittm.) and *Solanum microdontum* (Bitter) also grow sympatrically (Fig. 1). *Solanum kurtzianum* is a diploid species (2*n* = 2*x* = 24) that grows on four Argentinean provinces, along a transect approximately 900 km long (Hijmans et al. 2002; Fig. 2). It is adapted to dry regions within the Monte phytogeographical province (Cabrera 1976) with an annual average temperature of 13°C and an annual precipitation of 314 mm (Hijmans et al. 2002). The distribution range of *S. microdontum* extends from northern Argentina to southern Bolivia over a distance of about 1300 km (Hijmans et al. 2002; Fig. 2). It grows typically in the rain forest, corresponding to the Cabrera's (1976) phytogeographical province of the Yungas, although it extends further south than the limits of the rain forest in La Rioja province, where it grows in wet shady places with an annual average temperature of 14.5°C and an annual precipitation of 639 mm (Hijmans et al. 2002). Most of the populations of *S. microdontum* are diploid, although in the extreme southern end of its range, which corresponds to the locality of Guanchín, only triploid (2*n* = 3*x* = 36) populations have been recorded (Okada and Hawkes 1978; Okada 1981). *Solanum* × *rechei* has a very narrow distribution, and it seems to be better adapted to dry places with an annual average temperature of 12.8°C and an annual precipitation of 242 mm (Hijmans et al. 2002).

*Solanum* × *rechei* is a recently formed hybrid showing instabilities such as plants with severe floral abnormalities and low pollen fertility. Its hybrid origin was first suggested by Hawkes and Hjerting (1969). Okada and Hawkes (1978) further tested its hybridity by field studies, reciprocal synthetic reconstructions of the hybrid, analyses of morphological characters, chromosome counts, and pollen stainability. Clausen and Spooner (1998) confirmed the hybrid origin of *S*. × *rechei* performing RFLP analysis on accessions of the hybrids and the two parental species. The aim of this work was to study the basis of the novel variability originated by a recent interspecific hybridization event. For this purpose, we analyzed the epigenetic, genetic, and morphologic variability of wild populations and germplasm bank maintained accessions of three sympatric *Solanum* species, the natural hybrid *S*. × *rechei,* and its parental species *S. kurtzianum* and *S. microdontum*. We found that morphologic variability correlated better with epigenetic than with genetic variability. Additionally, a Bayesian approach showed that *S*. × *rechei* individuals established novel epigenetic patterns, different from those of the parental species, whilst maintaining genetically admixed genomes. These findings suggest that epigenetic variation may play an important role in the stabilization and evolution of this hybrid.

## Materials and Methods

### Plant material

The study was performed on two types of materials: (i) Plants from wild populations of *S. kurtzianum, S. microdontum,* and *S*. × *rechei* sampled as tubers in 2008 and 2010 at the department of Chilecito, La Rioja; and (ii) accessions of these species provided by the Potato and Forages Germplasm Bank Instituto Nacional de Tecnología Agropecuaria, Balcarce, Argentina (PFGB-INTA; Fig. 2 and Table 1). The study was carried out over two years. In the first year, tubers collected by the authors were sown in pots, and seeds provided by the PFGB were germinated in Petri dishes and then transplanted into pots. In the second year, all plant materials were grown from the tubers obtained in the previous season; thus, all genotypes were bred at the same time under uniform conditions in an insect-proof screen house.

**Table 1:** Plant material used in morphological and molecular analyses

Source	Province/Locality	Population code	Latitude	Longitude	Altitude (m)	Putative taxon	Number of plants	Molecular analysis	Morphological analysis	Flower phenotype
Author's Collection (2008)	La Rioja/Guanchín	GV02	29°10′21″S	67°39′02″W	1774	*S*. × *rechei*	3	Yes	Yes	N
		GV04	29°09′47″S	67°39′29″W	1818	*S*. × *rechei*	3	Yes	Yes	N
		GV05	29°09′46″S	67°39′32″W	1823	*S*. × *rechei*	2	Yes	Yes	N
		NN	29°09′46″S	67°39′30″W	1824	*S*. × *rechei*	4	Yes	Yes	SA
		GV07	29°09′44″S	67°39′31″W	1831	*S. microdontum*	6	Yes	Yes	N
		GV08	29°09′44″S	67°39′32″W	1829	*S*. × *rechei*	5	Yes	Yes	A
		GV09	29°09′42″S	67°39′38″W	1826	*S*. × *rechei*	6	Yes	Yes	A
		GV11	29°09′39″S	67°39′40″W	1841	*S*. × *rechei*	1	Yes	Yes	A
		GV12	29°09′42″S	67°39′25″W	1838	*S*. × *rechei*	3	Yes	Yes	N
	La Rioja/Cuesta de Miranda	LR1	29°25′59″S	67°49′57″W	1556	*S. kurtzianum*	1	Yes	No	N
		LR2	29°26′00″S	67°49′56″W	1557	*S. kurtzianum*	3	Yes	No	N
		LR3	29°25′59″S	67°49′55″W	1557	*S. kurtzianum*	3	Yes	No	N
	La Rioja/Cuesta de Guanchín	CG	29°11′00″S	67°35′59″W	1555	*S. kurtzianum*	3	Yes	No	N
Author's Collection (2010)	La Rioja/Guanchín	CMM	29°09′35″S	67°39′44″W	1845	*S. microdontum*	4	Yes	No	N
		CMK	29°09′36″S	67°39′42″W	1850	*S. kurtzianum*	6	Yes	No	N
		CMR	29°09′34″S	67°39′50″W	1846	*S*. × *rechei*	3	Yes	No	SA
Potato and Forages Germplasm Bank, INTA Balcarce	Mendoza/Las Heras	4544	32°33′S	68°58′W	680	*S. kurtzianum*	2	Yes	Yes	N
		4548	32°26′S	68°56′W	1600	*S. kurtzianum*	3	Yes	Yes	N
		4505	32°24′S	68°52′W	1185	*S. kurtzianum*	3	Yes	Yes	N
		4549	32°25′S	68°57′W	1660	*S. kurtzianum*	3	Yes	Yes	N
		4552	32°32′S	69°02′W	2330	*S. kurtzianum*	3	Yes	Yes	N
	Catamarca/Andalgalá	4631	27°19′S	66°39′W	2030	*S. microdontum*	5	Yes	Yes	N
	Salta/Santa Victoria	5902	22°08′S	65°02′W	2900	*S. microdontum*	3	Yes	Yes	N
	La Rioja/Chilecito	4572	29°13′S	67°39′W	1820	*S*. × *rechei*	1	No	Yes	A
		4574	29°12′S	67°39′W	1880	*S*. × *rechei*	2	Yes	Yes	A
		4583	29°08′S	67°37′W	1520	*S*. × *rechei*	1	Yes	Yes	A
Total		26					82			

N = Normal; SA = Slightly Abnormal; A = Abnormal.

### Morphological analysis

Thirty qualitative and quantitative characters were measured in three plants per accession once flowering had started (Table 2). Morphological measurements were performed on the seventh true leaf from the base of the plant and on the first inflorescence to appear. To test for hybridity based on morphological intermediacy, the “character count procedure” was performed as described by Wilson (1992). This analysis consists in tabulating intermediate and non-intermediate character states in the putative hybrid respect to its parents. To determine whether differences in characters were statistically significant, univariate analysis of variance (ANOVAs) were performed for each quantitative character and chi-square tests were employed for qualitative characters using STATGRAPHICS Centurion XVI software (StatPoint Technologies, Warrenton, VA). Only characters that differed between the parents were taken into account, and a one-sided sign test (Zar 1984) of intermediate versus non-intermediate characters was applied to judge whether the coalescence of intermediate character states was too improbable to represent divergence in the same characters and in the same direction. Also a principal coordinates analysis (PCoA) was carried out, and a minimum spanning tree (MST) was superimposed. As we had qualitative and quantitative variables, the Gower dissimilarity coefficient (Gower 1971) was used to generate a distance matrix in the PCoA. The operational taxonomic unit (OTU) was the average of three plants. A cluster analysis was performed using the same distance matrix and UPGMA (Unweighted Pair-Group Method with Arithmetic Averaging) as linkage method. R software 2.15.1 (R Core Team 2012) was used to perform the one-sided sign test in the character count procedure and to obtain the Gower dissimilarity matrix. The NTSYS-pc 2.11 software (Rohlf 1998) was employed for PCoA and cluster analysis.

**Table 2:** Morphological characters measured in *Solanum kurtzianum* (ktz), *Solanum microdontum* (mcd), and *Solanum* × *rechei* (rch) and test for intermediacy of *S*. × *rechei*

		Mean (SD)/Median^1^			ANOVA/chi-square test^2^			
				
Morphological character		ktz	mcd	rch	ktz–mcd	ktz–rch	mcd–rch	Intermediacy
1.	Leaf length (mm)	172.8 (28.9)	144.8 (27.2)	203.5 (27.4)	**	**	**	No
2.	Leaf width (mm)	142.8 (21.8)	98.8 (24.7)	185.3 (33.5)	**	**	**	No
3.	Lateral leaflet length (mm)	69.4 (10.1)	29.0 (21.2)	91.9 (17.9)	**	**	**	No
4.	Lateral leaflet width (mm)	34.2 (5.6)	19.1 (10.8)	47.2 (10.6)	**	**	**	No
5.	Terminal leaflet length (mm)	68.7 (6.7)	124.4 (25.4)	104.4 (28.8)	**	**	*	Yes
6.	Terminal leaflet width (mm)	38.5 (5.8)	70.8 (17.5)	63.8 (22.3)	**	**	ns	No
7.	Number of lateral leaflets (number of pairs)	8.7 (1.5)	0.7 (1.1)	6.7 (1.4)	**	**	**	Yes
8.	Number of intercalar leaflets	7.3 (7.7)	0.0 (0.0)	4.6 (3.3)	**	**	**	Yes
9.	Stem diameter (mm)	4.8 (1.0)	8.2 (1.9)	7.8 (1.1)	**	**	ns	No
10.	Corolla shape: ratio L/b	0.5 (0.1)	0.9 (0.3)	0.8 (0.3)	**	**	ns	No
11.	Corolla diameter (mm)	24.4 (2.8)	24.3 (2.7)	21.8 (2.9)	ns	**	**	–
12.	Distance corolla center – lobule apex (mm)	14.5 (1.7)	15.6 (1.9)	13.8 (1.3)	**	*	**	No
13.	Distance corolla center – lobule base (mm)	9.9 (1.4)	8.7 (1.6)	8.0 (1.9)	**	**	ns	No
14.	Number of petals	5.0 (0.1)	5.0 (0.1)	5.1 (0.2)	ns	ns	ns	–
15.	Style length (mm)	10.8 (1.0)	9.8 (0.6)	10.8 (0.8)	**	ns	**	No
16.	Anther length (mm)	5.4 (0.5)	6.1 (0.4)	5.7 (0.8)	**	*	*	Yes
17.	Calyx lobule length (mm)	2.6 (0.5)	5.1 (2.0)	4.4 (2.1)	**	**	ns	No
18.	Calix acumen length (mm)	1.3 (0.6)	1.5 (1.3)	1.9 (1.2)	ns	**	ns	–
19.	Number of tubers	12.9 (8.4)	13.4 (8.0)	11.9 (5.3)	ns	ns	ns	–
20.	Weight of the largest tuber (g)	9.0 (5.6)	11.3 (8.6)	13.1 (8.5)	ns	**	ns	–
21.	Weight of all tubers (g)	30.2 (16.1)	46.8 (23.9)	41.6 (16.0)	**	**	ns	No
22.	Terminal leaflet base [(1) acute (2) rounded (3) cordate (4) asymmetric]	2.5	3	3	ns	ns	ns	–
23.	Terminal leaflet apex [(1) acuminate (2) acute (3) obtuse]	1.5	2.5	2	**	**	**	Yes
24.	Wing size [(1) absent (2) small (3) large]	1	2	2	**	**	ns	No
25.	Stem pubescence [(1) abundant (2) moderate (3) absent]	3	3	3	ns	ns	ns	–
26.	Stem pigmentation [(1) green (2) green w/little purple (3) purple w/little green (4) purple]	2.5	1.5	3	**	**	**	No
27.	Leaf pubescence [(1) abundant (2) moderate (3) absent]	3	1	2	**	**	**	Yes
28.	Calyx pubescence [(1) abundant (2) moderate (3) absent]	3	1	2	**	**	**	Yes
29.	Corolla pigmentation [(1) white (2) white w/light purple star (3) white w/dark purple star]	3	1	1	**	**	ns	No
30.	Tuber pigmentation [(0) absent (1) slight (2) strong]	0	0	0	ns	ns	*	–
								I (7): N (15)

Significance levels: ** = *P* < 0.01; * = *P* < 0.05; ns = *P* > 0.05.

1 For quantitative characters (1–21), mean and standard deviation (SD) were calculated. For qualitative characters (22–30), the median was obtained.

2 Univariated ANOVA tests were performed for quantitative characters and chi-square tests for qualitative characters.

### Amplified fragments length polymorphism (AFLP) analysis

DNA was extracted from fresh leaves following Dellaporta et al. (1983) with an intermediate treatment with RNase. After quantification of DNA concentration by spectrophotometry (GeneQuant RNA/DNA Calculator, Pharmacia Biotech), samples were diluted to a concentration of 100 ng·μL^−1^.

To assess genetic variability in the evaluated material, an AFLP analysis was performed as described by Vos et al. (1995). *Eco*RI and *Mse*I were used as the rare and frequent cutter, respectively. The restriction reaction contained 2 units of *Eco*RI, 2 units of *Mse*I, 1.25 μL of buffer 2 (NEB), 100 ng·μL^−1^ BSA, and 250 ng of DNA in a final volume of 12.5 μL and was incubated for 3 h at 37°C. The ligation reaction contained 5 pmol of *Eco*RI adaptors, 50 pmol of *Mse*I adaptors, 1.25 μL T4 DNA ligase buffer (Promega), 0.75 units of T4 DNA ligase (Promega, Madison, WI), and 6.25 μL of the digested products in a final volume of 12.5 μL. Adaptors were ligated for 3 h at 20°C. Preselective amplification was accomplished using [*Eco*RI + A] and [*Mse*I + C] primers. Each PCR contained 40 ng of each primer, 400 μmol of dNTPs, 1× Taq DNA polymerase buffer, 1 unit of *Taq* DNA polymerase (Invitrogen), and 1 μL of the ligation products in a final volume of 25 μL. The amplification profile was as follows: 20 cycles at 94°C for 30 s, 56°C for 1 min, and 72°C for 1 min. The pre-selective PCR products were diluted 1:3 with sterile ultra-pure H_2_O. Selective amplification was performed with fluorescence-labeled *Eco*RI-selective primers and unlabeled *Mse*I selective primers (Table 3). The reaction contained 0.125 μmol·L^−1^ of [*Mse*I + 3] primers, 0.125 μmol·L^−1^ of [*Eco*RI + 3] labeled primers, 200 μmol·L^−1^ of dNTPs, 1× *Taq* DNA polymerase buffer (Invitrogen), 0.5 units of *Taq* DNA polymerase (Invitrogen), and 5 μL of the diluted pre-selective product in a final volume of 20 μL. The PCR protocol was as follows: 14 cycles at 94°C for 30 s, 65°C for 30 s (decreasing 0.7°C per cycle), and 72°C for 1 min, followed by 23 cycles at 94°C for 30 s, 56°C for 30 s, and 72°C for 1 min.

**Table 3:** AFLP and MSAP adaptors and primers used in this study and fragments obtained

	Sequences	Number of fragments
AFLP oligonucleotides		
Adaptors		
*Eco*RI	5′-AATTGGTACGCAGTCTAC	
	5′-CTCGTAGACTGCGTACC	
*Mse*I	5′-TACTCAGGACTCAT	
	5′-GACGATGAGTCCTGAG	
Preselective primers		
*Eco*RI + A	5′-AGACTGCGTACCAATTCA	
*Mse*I + C	5′-GACGATGAGTCCTGAGTAAC	
Selective primer combinations (*Eco*RI+3 and *Mse*I+3)		
*Eco*RI + ACG/*Mse*I + CTG		137
*Eco*RI + ACG/*Mse*I + CTC		64
*Eco*RI + ACA/*Mse*I + CTC		197
*Eco*RI + AAG/*Mse*I + CAC		191
*Eco*RI + ACA/*Mse*I + CTG		73
*Eco*RI + AAG/*Mse*I + CTG		170
	Total:	832
MSAP oligonucleotides		
Adaptors		
*Eco*RI	5′-CTCGTAGACTGCGTACC	
	5′-AATTGGTACGCAGTCTAC	
*Hpa*II – *Msp*I	5′-GACGATGAGTCTCGAT	
	5′-CGATCGAGACTCATC	
Preselective primers		
*Eco*RI + 0	5′-GACTGCGTACCAATTC	
*Hpa*II – *Msp*I + A	5′-ATGAGTCTCGATCGGA	
Selective primer combinations (*Eco*RI+3 and *Hpa*II – *Msp*II+3)		
*Eco*RI + AGA/*Hpa*II – *Msp*I + ATC		46
*Eco*RI + ACA/*Hpa*II – *Msp*I + AAT		46
*Eco*RI + ACA/*Hpa*II – *Msp*I + ATC		43
*Eco*RI + AAC/*Hpa*II – *Msp*I + ATC		40
*Eco*RI + AAC/*Hpa*II – *Msp*I + AAT		57
	Total:	232

After selective amplification, 1 μL of each sample was mixed with 8.5 μL Hi-Di formamide and 0.5 μL of the size standard Genescan 500HD ROX (Applied Biosystems, Foster City, CA). Samples were then denatured at 90°C for 3 min and were run on an Applied Biosystems Genetic Analyzer 3130. Semi-automated scoring was performed on the resulting electronic profiles using GeneMapper version 3.7 (Applied Biosystems).

### Methylation-sensitive amplified polymorphism (MSAP) analysis

**Figure 3:**
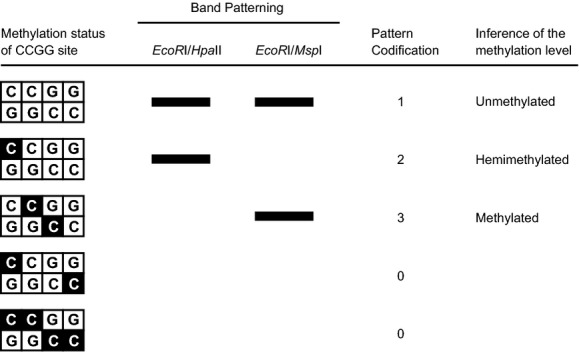
Methylation-sensitive amplified polymorphism (MSAP) band patterning determined by *Hpa*II/*Msp*I isoschizomers and correspondence with methylation status of CCGG sites. Black squares indicate methylated cytosines.

Epigenetic variability was estimated by MSAP analysis, which characterize the methylation pattern at anonymous 5′-CCGG sequences from random genomic DNA. The MSAP procedure was performed according to Xiong et al. (1999), with slight modifications. This is an adaptation of the original AFLP protocol, used to detect methylation polymorphism. It substitutes the frequent cutter enzyme *Mse*I by *Hpa*II and *Msp*I, while the rare cutter remains unchanged. *Hpa*II and *Msp*I are isoschizomers which recognize the same tetranucleotide restriction site (5′-CCGG), but have different sensitivities to methylation at cytosines. *Hpa*II is sensitive to full methylation (both strands methylated) of either cytosine but cleaves the hemimethylated external cytosine, whereas *Msp*I is sensitive only to methylation of the external cytosine. In this technique, the presence of the fragments in both profiles indicates a non-methylated site, while the absence of these fragments in both amplifications is ambiguous. It could be due to complete site methylation or to variation in the nucleotide sequence (Fig. 3).

In the first restriction assay, 700 ng of genomic DNA was digested with 10 U of *Eco*RI (Promega) with 100 ng·μL^−1^ BSA and 1× buffer H (Promega) in a final volume of 20 μL. To ensure complete digestion, incubation was performed at 37°C over night. The digest was split into two equal volumes and 10 U of *Hpa*II (Promega) and 10 U of *Msp*I (New England Biolabs) were added to each half, along with 100 ng·μL^−1^ BSA and the appropriate buffers in a final volume of 20 μL for the second digestion. Ten μL of the digested solution was ligated with 25 pmol of each *Eco*RI and *Hpa*II/*Msp*I adaptors, using 0.75 units of T4 DNA ligase (Promega) in a final volume of 20 μL. Adaptors were ligated for 3 h at 15°C. Pre-amplification was performed using 1 μL of the ligation products and 4 pmol of the [*Eco*RI + 0] and [*Hpa*II/*Msp*I + A] primers in a final volume of 20 μL containing 1× PCR buffer, 0.1 mmol·L^−1^ dNTPs, and 1 U *Taq* polymerase (Invitrogen). The pre-amplification products were diluted 1:3, and 1 μL was used in the selective amplification reaction with the *Eco*RI- and *Hpa*II/*Msp*I-selective primers (Table 3) in a final volume of 20 μL. The other components were the same as the pre-amplification reactions. In the pre-amplification as well as in the amplification, PCR programs were the same as in the AFLP protocol. The final amplification products were separated by electrophoresis on a 6% denaturing polyacrylamide gel, silver stained following Benbouza et al. (2006) and scanned for manual scoring.

### Data analysis

In the AFLP analysis, a binary matrix was constructed scoring fragments as present (1) or absent (0). For the MSAP analysis, two different matrices were built: (i) a binary matrix with methylation-insensitive polymorphisms, loci where the fragments were either present or absent in both *Eco*RI/*Hpa*II and *Eco*RI/*Msp*I digests for all the genotypes; and (ii) a methylation-sensitive polymorphisms matrix, with fragments that differed in the presence/absence patterns between the *Eco*RI/*Hpa*II and the *Eco*RI/*Msp*I digests in at least one genotype. In this case, patterns were codified from 0 to 3 (Fig. 3), and then, this codification was converted into a binary matrix for presence (1) or absence (0) of the particular pattern. A general methylation level was calculated for each species by computing the percentage of methylated, hemimethylated, and unmethylated loci, determined by patterns 3, 2, and 1, respectively. As absence of bands (pattern 0) could be due to methylation of external cytosines as well as variations in the nucleotide sequence, they were not considered as methylated loci.

For both AFLP and MSAP procedures, a subset of 23 samples were replicated starting from the same DNA extractions, to calculate the error rate of the techniques. Loci with different patterns on replicates were excluded from analysis as possible methodological artifacts.

The total number of fragments (loci) for AFLP and MSAP was computed for each of the three species. Common and species-specific fragments were identified, and the mean number of fragments per individual was also calculated. Based on MSAP data, percentages of methylated, hemimethylated, and unmethylated loci were determined for each species and for a combination of *S. microdontum* and *S. kurtzianum* (mid-parental value). ANOVAs were performed to compare fragments per individual and methylation percentages between species using STATGRAPHICS Centurion XVI software.

### Population structure

Multivariate analyses were carried out separately for genetic and epigenetic data sets. PCoA and cluster analyses were performed to determine relationships among individuals based on genetic and epigenetic polymorphism. Similarity matrices were generated based on the Dice coefficient, and the UPGMA linkage method was used for dendrogram construction. Also, the Mantel test was carried out to contrast genetic, epigenetic, and morphological matrices. For this, matrices containing shared individuals were constructed and similarity matrices, and tree topologies were compared using 1000 permutations. PCoA, cluster analysis, and the Mantel test were performed with NTSYS-pc 2.11, and for bootstrapping analysis, the WinBoot program was used (Yap and Nelson 1996). Genetic and epigenetic distance matrices were obtained based on the corresponding Dice similarity matrices. For this, each similarity value was transformed into distance value (*d* = 1–*s*), and a mean distance was calculated for each species. ANOVAs were carried out to compare genetic and epigenetic variability among species using STATGRAPHICS Centurion XVI software.

### Bayesian clustering

A model-based clustering method was used for inferring population structure using allele frequencies. We used the program STRUCTURE 2.3 (Pritchard et al. 2000) to analyze AFLP and MSAP data sets. Dominant marker data were entered by coding both alleles as (1) when the fragment was present and both as (0) when the fragment was absent, specifying (0) as a recessive allele for all loci as described by Falush et al. (2007). Estimates for the log likelihood were obtained using the admixture model and the assumption that the allele frequencies are correlated. The log likelihood was estimated from 10 replicate runs at each *K*, ranging from *K* = 1 to *K* = 6. For each run, we used a burn-in of 25,000 cycles and a data run of 100,000 cycles. For selecting the optimal value of K, similarity coefficients (Rosenberg et al. 2002) and ΔK values (Evanno et al. 2005) were calculated using STRUCTURE-SUM (Ehrich et al. 2007) with the R software 2.15.1.

### Detection of epiloci under selection

The MSAP data set was analyzed to identify candidate epiloci under natural selection using the Bayesian method proposed by Foll and Gaggiotti (2008) and implemented in the program BayeScan 2.1 (http://cmpg.unibe.ch/software/bayescan). Assuming that allele frequencies within populations follow a Dirichlet distribution, BayeScan calculates locus-population F_ST_ coefficients. Selection is introduced by decomposing locus population F_ST_ coefficients into a population-specific component β (beta), shared by all loci, and a locus-specific component α (alpha), shared by all the populations using a logistic regression. Departure from neutrality at a given locus is assumed when the locus-specific component is necessary to explain the observed pattern of diversity (α significantly different from 0). A positive value of alpha suggests diversifying selection, whereas negative values suggest balancing or purifying selection. This leads to two alternative models for each locus, including or not the alpha component to model selection. BayeScan implements a reversible-jump MCMC algorithm to estimate the posterior probability of each one of these models. Posterior odds (PO) are simply the ratio of posterior probabilities and indicate how more likely the model with selection is compared with the neutral model (Foll and Gaggiotti 2008).

We used 20 pilot runs of 5000 iterations to adjust acceptance rate for each parameter. A burn-in of 50,000 iterations was employed to attain convergence before starting the sampling. The sample size was set to 5000 with a thinning interval of 10, resulting in a total chain length of 100,000 iterations. The loci were ranked according to their estimated posterior probability, and all loci with a value greater than 0.91 were retained as outliers. This corresponds to log_10_(PO) >1, which provides decisive support for acceptation of the model according to Jeffreys' scale of evidence (Jeffreys 1961).

### Isolation, sequence, and bioinformatic analysis of MSAP fragments

Fragments of interest were excised from the gel and heated at 95°C for 20 min in 100 μL of ultrapure water. The eluted DNA fragment was re-amplified with the same primers as the MSAP reaction. The PCR protocol was as follows: 1 cycle at 94°C for 3 min, 35 cycles at 94°C for 1 min, 58°C for 30 s, and 72°C for 1 min, and a final extension step at 72°C for 20 min. The PCR products were resolved in a 1.2% agarose gel, sliced, and purified with Wizard® SV Gel and PCR Clean-Up System (Promega) following the manufacturer's protocol. Direct sequencing of the fragments was performed by Sanger technology in an Applied Biosystems Genetic Analyzer 3130XL. Sequences were edited and analyzed with BioEdit 7.0.9 (Hall 1999).

Analysis of DNA similarity was carried out using the BLASTn packages implemented at The Institute for Genomic Research (TIGR; http://www.jcvi.org/; http://blast.jcvi.org/euk-blast/plantta_blast.cgi).

## Results

### Collection of plant material

We collected plant material from the hybridization area, in the locality of Guanchín (La Rioja), in two opportunities. In the 2008 expedition, we found that the most common species was the hybrid *S*. × *rechei*. Several populations of this species were identified growing in modified environments such as backyards, roadsides, and under walnut (*Juglans sp*.) and horticultural orchards. The parental species, *S. kurtzianum* and *S. microdontum*, were not present in these disturbed areas. We also explored natural environments for the presence of the three species. In the gorge of the Pismanta River, along a transect approximately 1 km long, we sampled six populations of *S*. × *rechei* (Table 1). No populations of *S. kurtzianum* were observed, and we were able to found only one population of *S. microdontum* (GV07). Then, we explored several gorges in the road to Mina de Oro, and only one population of *S*. × *rechei* was sampled (GV12). We collected four populations of *S. kurtzianum* separated from the area of hybridization by a distance of about of 5 km (CG) and 50 km (LR1, LR2, and LR3). In 2010, we expanded the collection area along the gorge of the Pismanta River in a transect of about two kilometers long. Again, we found that the species with greater distribution was the hybrid *S*. × *rechei* and sampled one more population (CMR). In addition, we found and sampled one additional population of *S. microdontum* (CMM) and one population of *S. kurtzianum* (CMK) within the gorge of Pismanta River.

### Morphological variation and floral abnormalities in *S*. × *rechei*

We measured 30 qualitative and quantitative characters in three plants per accession once flowering had started (Table 2) to test for hybridity based on morphological intermediacy. There was superimposition in four (14, 19, 22, and 25) of 30 characters among the three species (Table 2). The parental species, *S. kurtzianum* and *S. microdontum*, showed no significant differences in four (11, 18, 20, and 30) additional characters.

For the character count procedure, only those that significantly differed between *S. kurtzianum* and *S. microdontum* were analyzed (22 of 30). Seven characters resulted intermediate and 15 resulted non-intermediate (Table 2); thus, the one-sided test of intermediate versus non-intermediate characters was not significant (*P* > 0.05), and the hypothesis of divergence could not be rejected. Six of the non-intermediate characters were transgressive in *S*. × *rechei* beyond the parental species.

**Figure 4:**
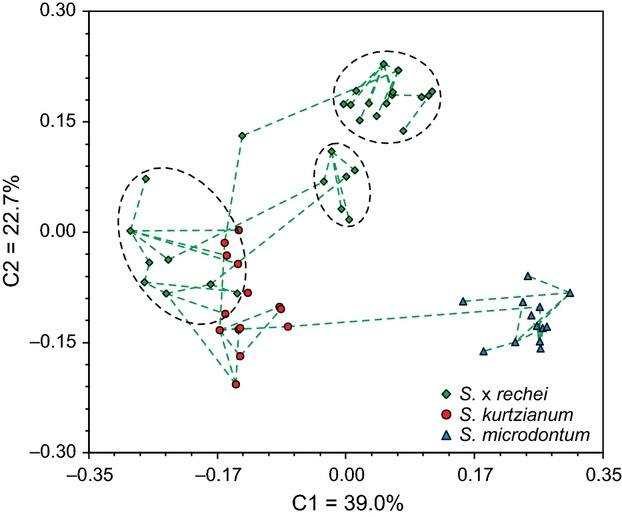
Principal coordinates analysis and minimum spanning tree of *Solanum kurtzianum*, *Solanum microdontum,* and *Solanum* × *rechei* based on 30 morphological characters.

Additionally, the association between the three species was determined by a multivariate analysis based on the 30 morphological characters. Figure 4 shows a plot of the first two coordinates of the PCoA, which accounted for more than 60% of the variability. Individuals identified as *S. microdontum* and *S. kurtzianum* by their morphotype were all gathered into two well-defined groups. On the other hand, individuals classified as *S*. × *rechei* formed three different groups: two located close to each other and between *S. kurtzianum* and *S. microdontum*, and a third group overlapped with *S. kurtzianum* group. In the cluster analysis, *S*. × *rechei* individuals were distributed in a main cluster and in a second cluster mixed with *S. kurtzianum* individuals, while *S. microdontum* formed a separated group (Fig. S1a).

Several *S*. × *rechei* accessions showed floral abnormalities (Table 1) that ranged from slight changes in the corolla form and symmetry to severe alterations such as rudimentary stamens and petals; homeotic changes like staminoid petals and carpelloid stamens (Fig. S2).

### AFLP and MSAP analyses

**Figure 5:**
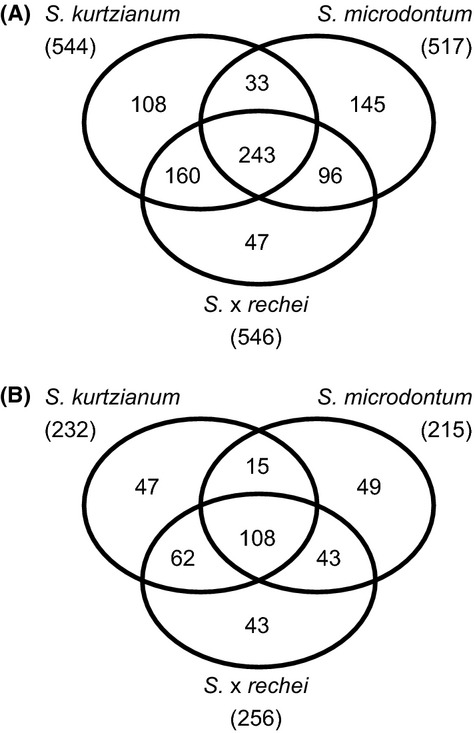
Venn diagrams showing the distribution of AFLP (A) and MSAP (B) fragments in *Solanum kurtzianum*, *Solanum microdontum,* and *Solanum* × *rechei*. In parenthesis, total number of different fragments (loci) per species.

The AFLP analysis with six primer combinations produced 832 fragments for the 81 individuals analyzed, 98% of which were polymorphic. The error rate based on replicated samples was 3.2%. The total number of fragments obtained was similar for each species, and the mean number of fragments per individual showed no significant differences among species (Fig. S3a). Of the 276 fragments shared by the parental species, 33 were absent in the hybrid *S*. × *rechei*. On the other hand, 47 fragments were found in *S*. × *rechei,* but not in the parental species. The distribution of fragments among the progenitor species and the hybrid is shown in Figure 5A.

**Figure 6:**
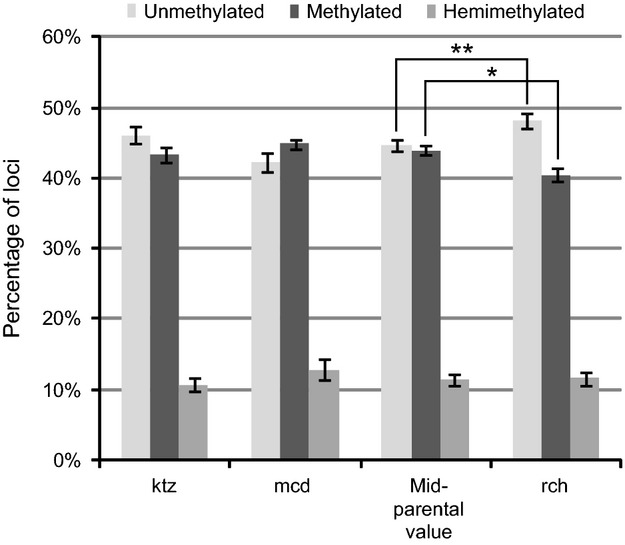
Percentage of methylated, hemimethylated, and unmethylated loci based on MSAP data for *Solanum kurtzianum* (ktz), *Solanum microdontum* (mcd), *Solanum* × *rechei* (rch), and a mid-parental value. Significance levels are **P < 0.01, *P < 0.05.

In the MSAP analysis, five primer combinations were used and a total of 231 loci were analyzed in 77 individuals. The error rate based on replicated samples was 3.4%. *Solanum* × *rechei* presented more fragments than the other two species, and the number of fragments per individual was significantly higher for *S*. × *rechei* than for *S. kurtzianum* (Fig. S3b). Thirty-two loci were scored as methylation-insensitive. The other 199 loci (86%) were considered methylation-sensitive and used to determine the epigenetic diversity. The percentage of unmethylated loci for *S*. × *rechei* (Mean = 48.0%) was significantly (*P* < 0.01) higher than that for the mid-parental value (Mean = 44.7%). The percentage of methylated loci for *S*. × *rechei* (Mean = 40.5%) was significantly (*P* < 0.05) lower than that for the mid-parental value (Mean = 43.9%). The difference in the hemi-methylation level was not statistically significant (*P* > 0.05; Fig. 6).

The Venn diagram in Figure 5B shows methylation patterns common to two or all three species and unique patterns for each species. The hybrid *S*. × *rechei* presented 43 exclusive patterns, not observed in the parental species, representing 12% of all patterns detected. Among them, 29 (8%) are unequivocally methylation changes; as for the same loci, the parental species showed the presence of the fragments, but with a different pattern respect to the hybrid. For the other 14 loci (4%), the parental species showed no fragments, which could indicate both a change in the methylation status in the hybrid (if the sites where hypermethylated in the parents) or a genetic variation.

### Genetic and epigenetic structure

**Figure 7:**
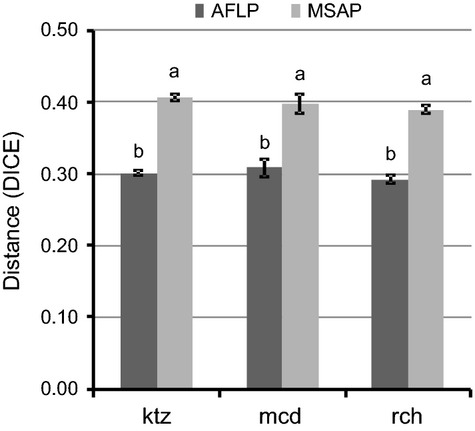
Genetic and epigenetic distances (Dice coefficient) of *Solanum kurtzianum* (ktz), *Solanum microdontum* (mcd), and *Solanum* × *rechei* (rch) based on AFLP and MSAP markers, respectively. Different letters indicate statistically significant difference at *P* < 0.01.

Genetic and epigenetic distances among individuals of the same species were used to calculate the genetic and epigenetic variability of each species. No significant differences in variability (genetic or epigenetic) were found between species (*P* > 0.05), although the comparison of genetic against epigenetic distances within species was statistically significant for the three species (*P* < 0.01; Fig. 7).

**Figure 8:**
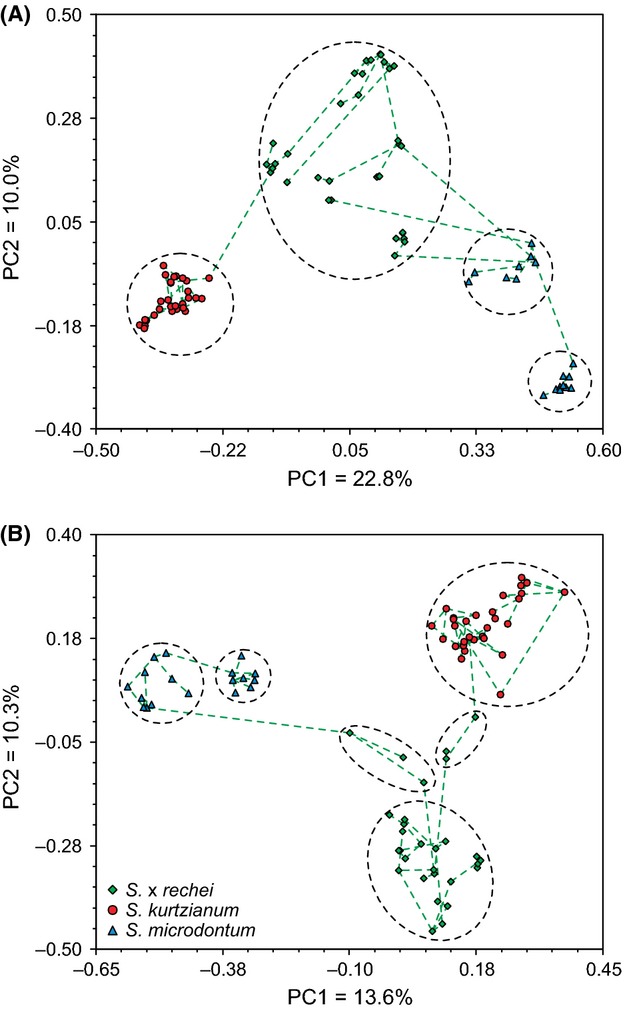
Principal coordinates analyses and minimum spanning tree of *Solanum kurtzianum*, *Solanum microdontum,* and *Solanum* × *rechei* individuals based on nuclear AFLP markers (A) and MSAP patterns (B).

The two-first principal coordinates of the PCoA with the AFLP and the MSAP data sets (Fig. 8A,B) accounted for 32.8% and 24% of the variance, respectively. Genetically, *S. kurtzianum* group showed a greater aggregation of the individuals than epigenetically. In the first case, all individuals were clustered together, while in the second case, genotypes from La Rioja separated from those accessions from Mendoza. *Solanum microdontum* behaved similarly in both studies, where plants collected in La Rioja province and the accessions from Catamarca and Salta provinces provided by the PFGB established two distinct subgroups. *Solanum* × *rechei* individuals in both plots were located between the parental species; although in the MSAP plot, they were considerably closer to *S. kurtzianum*. Additionally, in the AFLP analysis, the hybrid was dispersed in many subgroups, which corresponded to the different sampled natural populations, while in the MSAP analysis, the group was more condensed except for populations GV02 and CMR. In the cluster analysis, individuals were grouped similarly as in the PCoA (Fig. S1b,c).

The Mantel tests (Table 4) showed a higher correlation of morphological data with MSAP (*r* = 0.55, *P* < 0.01) than with AFLP (*r* = 0.38, *P* < 0.01). MSAP matrix holds a greater correlation with AFLP (*r* = 0.78) given that both are methodologically related (i.e., for two individuals to have the same methylation state at any given locus, they must first share the locus).

**Table 4:** Pairwise comparison of MSAP, AFLP, and morphology matrices with the Mantel test

	MSAP	AFLP	Morphology
MSAP	1	0.917	−0.628
AFLP	0.784 (77)	1	−0.513
Morphology	−0.550 (56)	−0.383 (56)	1

Below the diagonal: comparison between similarity matrices. Above the diagonal: comparison of tree topologies transformed to matrix values through cophenetic correlation. Numbers in parentheses indicate the number of accessions common to each comparison. 0 = no correspondence, 1 = perfect correspondence. *P* < 0.01 was obtained for all comparisons based on 1000 permutations test.

### Population structure

To choose the optimal number of groups for the STRUCTURE analysis, 10 runs were performed for each value of K from 1 to 6. For the AFLP data set, *K* = 2 and 3 presented similarity coefficients close to 1, which means that individuals were repeatedly assigned to the same groups in all runs. This behavior was also observed in the bar plots. With MSAP data, the similarity coefficient was 1 only for *K* = 3. Furthermore, the Δ*K* values suggested *K* = 2 as the optimal for AFLP and *K* = 3 for MSAP (Fig. S4).

**Figure 9:**
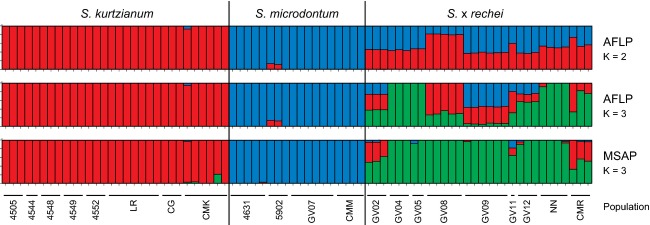
Bar plots showing genetic and epigenetic structure of *Solanum kurtzianum*, *Solanum microdontum,* and *Solanum* × *rechei* populations based on AFLP and MSAP data, respectively. Each accession is represented by a vertical line partitioned in K colors that represent the proportion of membership to each cluster. For AFLP, *K* = 2 and 3 and for MSAP, *K* = 3 are shown.

In Figure 9, the populations genetic structure for *K* = 2 and 3 and epigenetic structure for *K* = 3 are represented. Each vertical line corresponds to an individual, and each color symbolizes the relative percentage of membership to each cluster. The parental species, *S. kurtzianum* and *S. microdontum,* corresponded to one cluster each and showed negligible admixture levels for both AFLP and MSAP data. In contrast, *S*. × *rechei* differed greatly between its genetic and epigenetic structure. For AFLP data, when *K* = 2 was tested, and all *S*. × *rechei* individuals presented certain percentage of membership to both parental clusters, and for *K* = 3, some individuals were assigned to a third group, while the rest presented high levels of admixture with the parental groups. For MSAP data, most individuals of *S*. × *rechei* belonged to their own cluster.

### Detection of epiloci under selection

BayeScan analysis identified 24 MSAP epiloci under selection (or linked to selected loci), with “strong evidence” (log_10_(PO) > 1) on the Jeffreys' scale. Fifteen of these markers were pointed out “very strongly” to be under selection (log_10_(PO) > 1.5), and for seven of them, evidence was considered “decisive” (log_10_(PO) > 2; Fig. S5). All these epiloci were characterized by high F_ST_ coefficients and positive α values and are therefore interpreted as having been affected by divergent selection.

Four MSAP fragments with the highest F_ST_ coefficients were isolated and sequenced. Two sequences were of high quality and were used for BLAST search on the TIGR database. One MSAP fragment of 144 bp was similar (*E* value 1.6 × 10^−13^) to an EST (Expressed Sequence Tag) from *Solanum chacoense* (GeneBank Accession DN978520). The other fragment of 125 bp was similar (*E* value 4.9 × 10^−7^) to a sequence of *Solanum lycopersicum* (GeneBank Accession TA55928_4081) annotated as a pararetrovirus-like sequence reported in tobacco.

## Discussion

In the present work, we analyzed the genetic and epigenetic variation generated in the recently formed natural potato hybrid *S*. × *rechei* based on a genome-wide screening method. The hybrid nature of *S*. × *rechei* had been analyzed using morphological and molecular (RFLPs) approaches by Okada and Hawkes (1978) and Clausen and Spooner (1998), respectively. However, Clausen and Spooner (1998) used for the RFLP analysis the diploid accessions of the parental species *S. microdontum* from non sympatric regions northern of the hybridization area in the province of La Rioja, Argentina. Here, we studied plants of *S. microdontum* from two diploid accessions (accession number 4631 and 5902 from Catamarca and Salta provinces, respectively) used by Clausen and Spooner (1998), and in addition, we collected triploid accessions (M. S. Ferrer, C. F. Marfil, and R. W. Masuelli, unpublished results) of *S. microdontum* from Guanchín (La Rioja province) growing sympatrically with *S*. × *rechei*. In *S. microdontum*, PcoA analyses of genetic data showed intraspecific discontinuities, separating populations from La Rioja province in one group and the populations from Catamarca and Salta provinces in another group. However, these differences were not supported in the Bayesian STRUCTURE analysis. This result confirms that *S. microdontum* material used by Clausen and Spooner (1998) was representative of the parental populations.

As described by Marfil et al. (2009) for a different potato hybrid (*Solanum ruiz-lealii*), *S*. × *rechei* presented genotypes with different floral abnormalities, suggesting that recently formed hybrids may suffer genetic and epigenetic instability as a result of the interspecific hybridization event.

Besides the general additivity of the hybrid respect to the parental genomes, gain (5.6%) and loss (4%) of AFLP fragments were observed in the hybrid, possibly as a result of genome rearrangements induced by the interspecific hybridization. Similar results were described in interspecific hybrids of wheat (Liu et al. 1998), *Spartina* spp. (Salmon et al. 2005) and *Solanum* (Marfil et al. 2006). In the hybrid, the epigenetic modification was more significant than the genetic changes, showing twice (12%) the number of novel fragments in MSAP than in the AFLP analysis. The MSAP loci analyzed showed a general demethylation compared with the parental genomes. Plants of *S. kurtzianum* and *S. microdontum* had 43% and 45% methylated loci, respectively, while 40% of the loci from *S*. × *rechei* were methylated. Zhao et al. (2007), compared relative levels of cytosine methylation at the CCGG sites and observed demethylation in maize hybrids respect to their parental inbred lines. Previous studies have showed that chemical demethylation of the potato hybrid *S. ruiz-lealii* induced floral abnormalities similar to the phenotypes observed in natural populations (Marfil et al. 2009). In addition, early flowering, changes in leaf morphology, and up-regulation of miRNAs were observed in plants treated with the demethylating agent AzaC (Marfil et al. 2012). Additional evidence of genomewide demethylation associated with runaway transposition of retroelements comes from interspecific hybridization between two wallaby species (Waugh O'Neill et al. 1998) and from newly synthetic *Arabidopsis* allopolyploids that showed loss of cytosine methylation in retrotransposon-related elements (Comai et al. 2000).

Our study showed a high level of genetic and epigenetic polymorphism for the three taxa analyzed, where 98% and 99% of the AFLP and MSAP loci were polymorphic, respectively. For each of the studied taxa, the epigenetic variability was significantly higher than the genetic variability, based on Dice coefficient (Fig. 7). PcoA analyses of morphological, genetic, and epigenetic information differentiated the three taxa and grouped the hybrids in intermediate position between the two parental species (Figs. 4, 8). The Bayesian STRUCTURE analysis of the genetic data showed that *S*. × *rechei* accessions, except for accession GV08 and one individual from CMR and GV11, were assigned to a cluster with posterior probability lower than 0.5 (Fig. 9). These results reveal that *S*. × *rechei* individuals have different levels of genetic admixture, indicating a recent hybridization and introgression between *S. kurtzianum* and *S. microdontum*. Interestingly, pairwise comparison analysis among the three matrices showed that the morphological data correlated better (*r* = 0.550, *P* < 0.01) with the epigenetic than the genetic information (*r* = 0.383, *P* < 0.01; Table 4). These results suggest that, in a recently formed hybrid, the epigenetic variation influences the phenotypic outcome more than the genetic one. This conclusion was supported by the Bayesian STRUCTURE analysis of the genetic and epigenetic data. The genetic data showed the hybrid nature of *S*. × *rechei* with an estimated number of groups of 2 (*K* = 2) with individuals displaying admixture between *S. kurtzianum* and *S. microdontum*. Instead, the epigenetic information clearly assigned *S*. × *rechei* individuals to a third cluster (*K* = 3) with high probability (Fig. 9 and Fig. S4), with seven individuals of 30 with more than 10% of admixture. These results indicate that an epigenetic pattern was rapidly established in the hybrids differentiating them from the parental taxa. In allopolyploid orchids, *Dactylorhiza*, Paun et al. (2010) showed that the methylation patterns explain better the gene expression differences between allopolyploids than the genetic information. It is noteworthy to show that in *S*. × *rechei* the hybridization event rapidly established a novel epigenetic pattern, different from the parental species, which could modulate the regulatory networks and persist stably over many generations possibly through clonal propagation by tubers. According to our results, the adaptation of *S*. × *rechei* to grow as a weed in disturbed environments, such as along roadsides or under trees in orchards (Okada and Hawkes 1978), is better explained by the rapid setup, after the hybridization event, of an epigenetic pattern influencing phenotypic plasticity and adaptation to environmental changes. BayeScan analysis identified 24 epialleles under selection, seven of them with high F_ST_ values and therefore interpreted as having been affected probably by divergent selection. Nevertheless, it is possible that these epialleles may have arisen as a consequence of the hybridization event and have been maintained by clonal propagation. One of the sequenced epialleles was related to an endogenous pararetrovirus sequence reported in tobacco, possibly conferring heritable virus resistance (Mette et al. 2002). The authors found that the pararetrovirus sequence was persistently methylated in CNG and CG sites, and the methylation patterns were preserved in two tobacco species, the allotetraploid *Nicotiana tabacum* and the diploid *N. sylvestris*. These results suggest that the epiallele was selected in both species and conferred advantageous traits to tobacco and *N. sylvestris*. It is likely that the same epiallele was under selection in the potato species studied.

Changes in chromosome number could play a role in the origin of the variability naturally found in the hybrid populations. As the hybrids were probably originated by the hybridization of the diploid *S. kurtzianum* and triploid cytotypes of *S. microdontum*, some hybrid genotypes are likely to be aneuploids. However, Okada and Hawkes (1978) found only euploid (both diploid and triploids) hybrid cytotypes in nature. We are currently analyzing in natural and synthetic hybrid plants the occurrence of aneuploids and the effect on plant phenotypes. Preliminary results indicate that some hybrid plants are aneuploids (M. S. Ferrer, C. F. Marfil and R. W. Masuelli, unpublished results).

## Concluding Remarks

Hybridization is an important event in the evolution of the potato species, and several authors suggested that more than 10% of the *Solanum* species from section *Petota* are the result of hybridization. There is also an important amount of phenotypic plasticity in this section (Correll 1962; Hawkes and Hjerting 1969). Our results show that after hybridization, a novel epigenetic pattern is rapidly established and maintained through several generations allowing the adaptation of hybrids to new environments. The importance of epialleles in the evolutionary process is intriguing. We found that for the three species, the methylation polymorphism detected was higher than nuclear DNA variability; and in addition, a better correlation between epigenetic patterns and phenotypic characters was observed. According to Kalisz and Purugganan (2004), given that epiallelic phenotypes are more flexible than alleles produced by genetic mutation, it is possible that phenotypes produced by epialleles could explore novel environments and drive the adaptation of other genes in the short term. We strongly suggest that epigenetic variation, together with genetic variation, plays a relevant role in the evolution of the *Solanum* species section *Petota* and that the analysis of the epigenetic variation may shed some light on the intricate taxonomy and systematic relationships among species of this section.

## Data Accessibility

Location, AFLP, and MSAP data: Dryad entry doi:10.5061/dryad.q6s27.

## Supporting Information

**Figure S1.** Cluster analyses *of Solanum kurtzianum, Solanum microdontum and Solanum* × *rechei* based on morphological (a), AFLP (b) and MSAP (c) data. For the AFLP and MSAP dendrograms bootstrap values are shown above the branches.

**Figure S2.** Flower phenotypes of *Solanum × rechei* individuals.

**Figure S3.** Total number of fragments per species and mean number of fragments per individual obtained by AFLP (a) and MSAP (b) techniques.

**Figure S4.** Similarity coefficients and Delta K values for AFLP and MSAP datasets.

**Figure S5.** Scan to identify epiloci subject to selection using BayeScan.
