# Supplementary material for: Epigenetic patterns newly established after interspecific hybridization in natural populations of Solanum
Source: Ecol Evol. 2013 Sep 9;3(11):3764–79. doi: 10.1002/ece3.758 (PMC3810873; doi:10.1002/ece3.758)
Supplement: Supplementary file 1 [file ece30003-3764-SD1.pdf]

Fig. S1 Cluster analyses *of Solanum kurtzianum, Solanum microdontum and Solanum x rechei* based on morphological (a), AFLP (b) and MSAP (c) data. For the AFLP and MSAP dendrograms bootstrap values are shown above the branches.

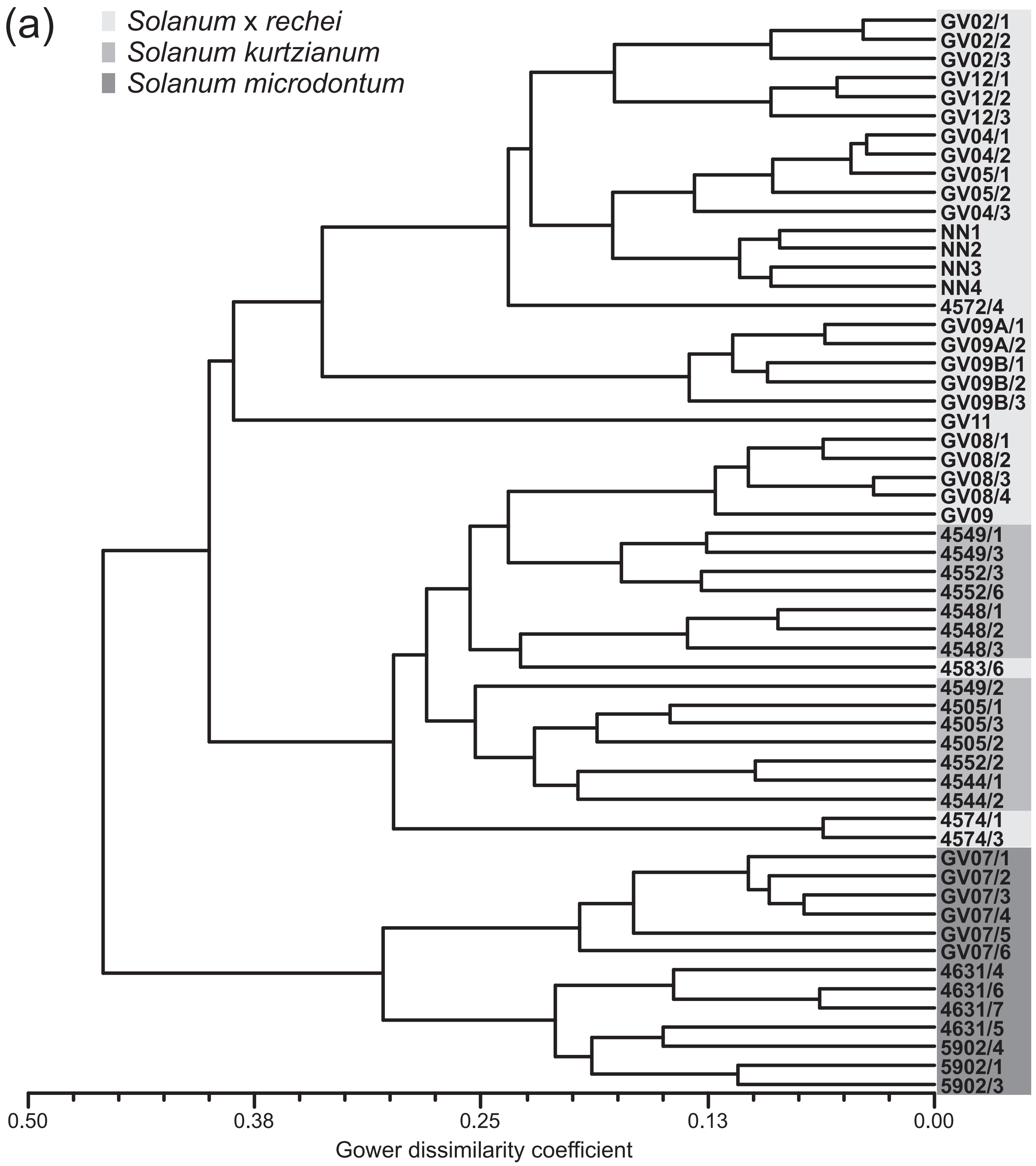

(b)

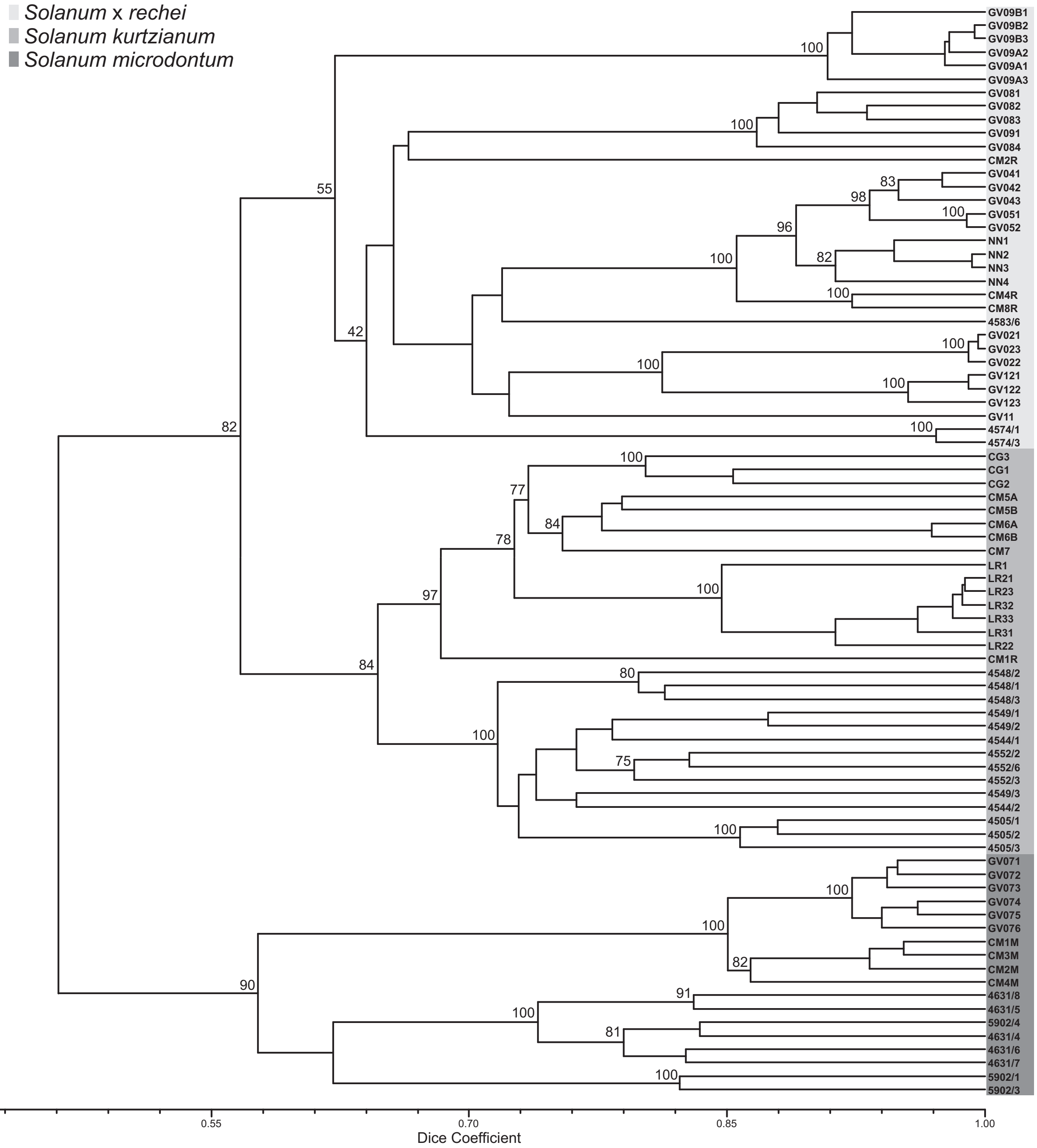

Solanum x rechei
Solanum kurtzianum
Solanum microdontum
GV09B1
GV09B2
GV09B3
GV09A2
GV09A1
GV09A3
GV081
GV082
GV083
GV091
GV084
CM2R
GV041
GV042
GV043
GV051
GV052
NN1
NN2
NN3
NN4
CM4R
CM8R
4583/6
GV021
GV023
GV022
GV121
GV122
GV123
GV11
4574/1
4574/3
CG3
CG1
CG2
CM5A
CM5B
CM6A
CM6B
CM7
LR1
LR21
LR23
LR32
LR33
LR31
LR22
CM1R
4548/2
4548/1
4548/3
4549/1
4549/2
4544/1
4552/2
4552/6
4552/3
4549/3
4544/2
4505/1
4505/2
4505/3
GV071
GV072
GV073
GV074
GV075
GV076
CM1M
CM3M
CM2M
CM4M
4631/8
4631/5
5902/4
4631/4
4631/6
4631/7
5902/1
5902/3
0.40
0.55
0.70
0.85
1.00
Dice Coefficient

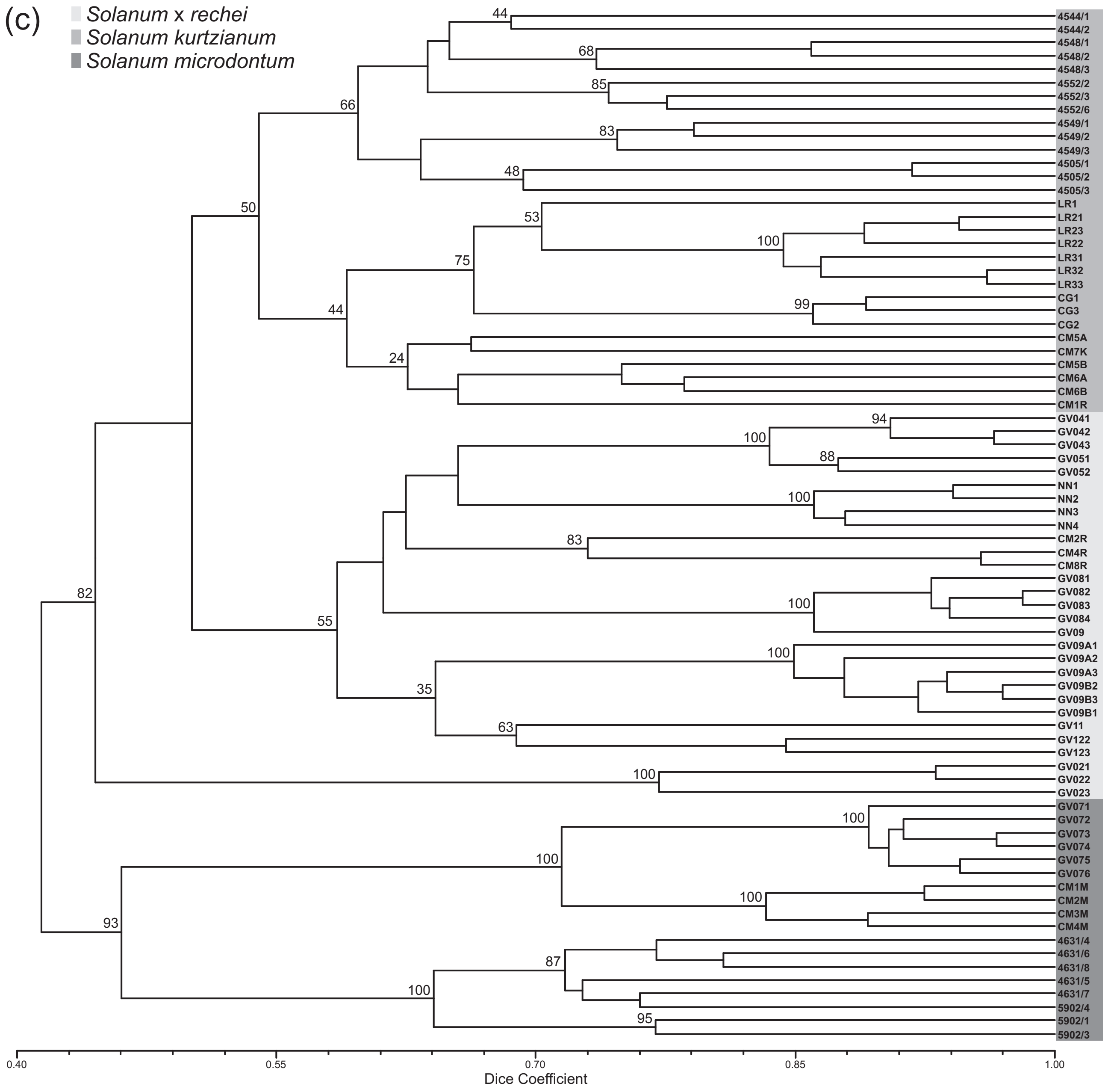
(c)
Solanum x rechei
Solanum kurtzianum
Solanum microdontum
4544/1
4544/2
4548/1
4548/2
4548/3
4552/2
4552/3
4552/6
4549/1
4549/2
4549/3
4505/1
4505/2
4505/3
LR1
LR21
LR23
LR22
LR31
LR32
LR33
CG1
CG3
CG2
CM5A
CM7K
CM5B
CM6A
CM6B
CM1R
GV041
GV042
GV043
GV051
GV052
NN1
NN2
NN3
NN4
CM2R
CM4R
CM8R
GV081
GV082
GV083
GV084
GV09
GV09A1
GV09A2
GV09A3
GV09B2
GV09B3
GV09B1
GV11
GV122
GV123
GV021
GV022
GV023
GV071
GV072
GV073
GV074
GV075
GV076
CM1M
CM2M
CM3M
CM4M
4631/4
4631/6
4631/8
4631/5
4631/7
5902/4
5902/1
5902/3
0.40
0.55
0.70
0.85
1.00
Dice Coefficient
