# Supplementary material for: Epigenetic patterns newly established after interspecific hybridization in natural populations of Solanum
Source: Ecol Evol. 2013 Sep 9;3(11):3764–79. doi: 10.1002/ece3.758 (PMC3810873; doi:10.1002/ece3.758)

Fig. S2 Flower phenotypes of *Solanum* x *rechei* individuals. (a), (b), (c) and (d), normal flowers from populations GV02, GV04, GV05 and GV12, respectively. From (e) to (g), flowers from population GV08. (e), flower with rudimentary stamens and dissected petals. (f), flower with dissected and extra number of petals and twisted style. (g), flower with extra number of carpels. (h), inflorescence with aborted flowers from population GV11. (i) and (j), flowers from population GV09. (i), flower with partially expanded petals and carpelloid stamens. (j), bottom view of the same flower from (I) showing the oversized and asymmetric calyx that hinders normal anthesis. (k) and (l), flowers from populations NN and CMR that resemble normal flowers, but with small and greenish stamens.

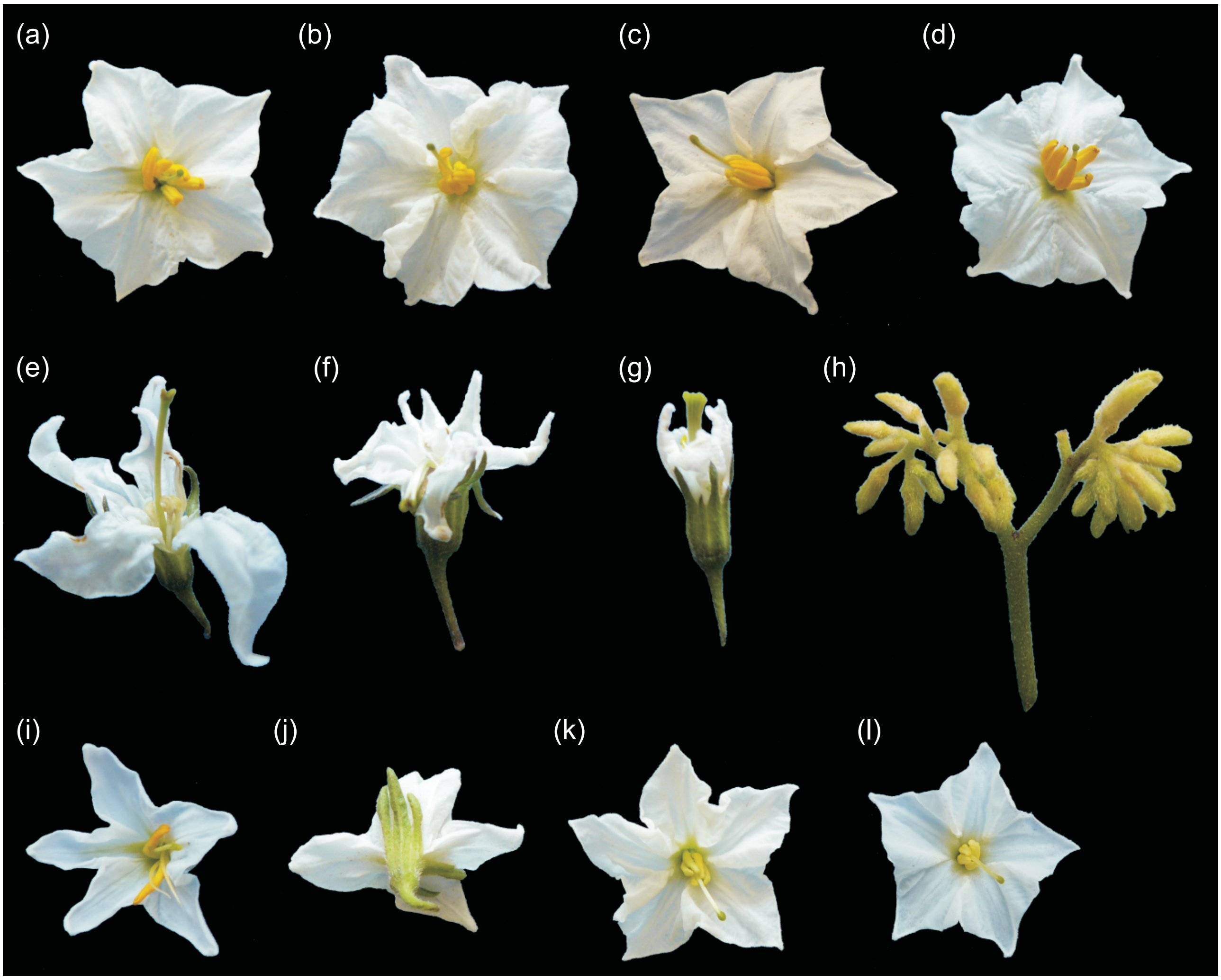

Supplement: Supplementary file 2 [file ece30003-3764-SD2.pdf]
