# Supplementary material for: Epigenetic patterns newly established after interspecific hybridization in natural populations of Solanum
Source: Ecol Evol. 2013 Sep 9;3(11):3764–79. doi: 10.1002/ece3.758 (PMC3810873; doi:10.1002/ece3.758)

Fig. S3 Total number of fragments per species and mean number of fragments per individual obtained by AFLP (a) and MSAP (b) techniques. *Different letters* indicate statistically significant difference at $P < 0.01$.

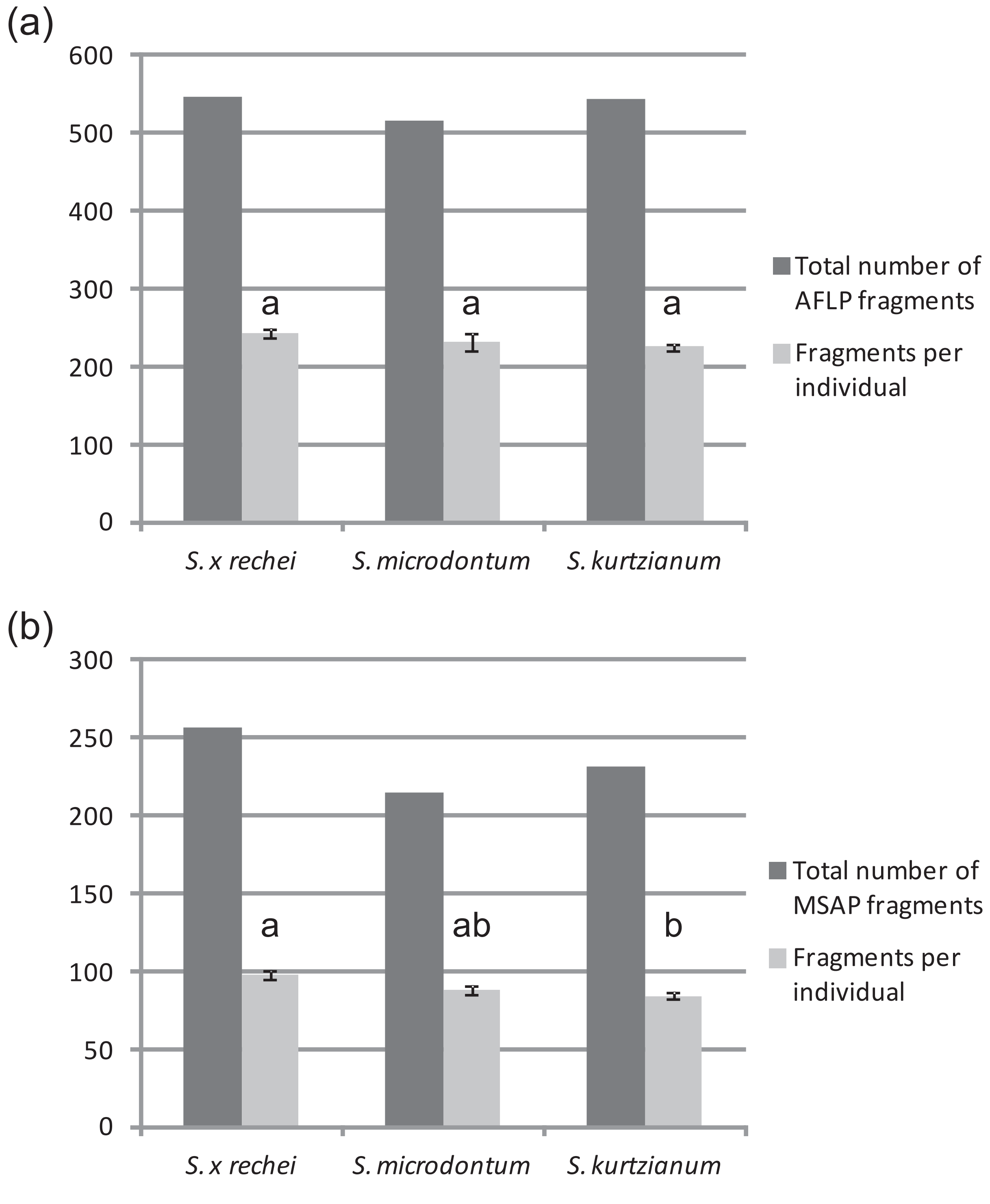

Supplement: Supplementary file 3 [file ece30003-3764-SD3.pdf]
