# Supplementary figures and images for: Epigenetic patterns newly established after interspecific hybridization in natural populations of Solanum
Source: Ecol Evol. 2013 Sep 9;3(11):3764–79. doi: 10.1002/ece3.758 (PMC3810873; doi:10.1002/ece3.758)

**Fig. S4** Similarity coefficients and Delta K values for AFLP and MSAP datasets.

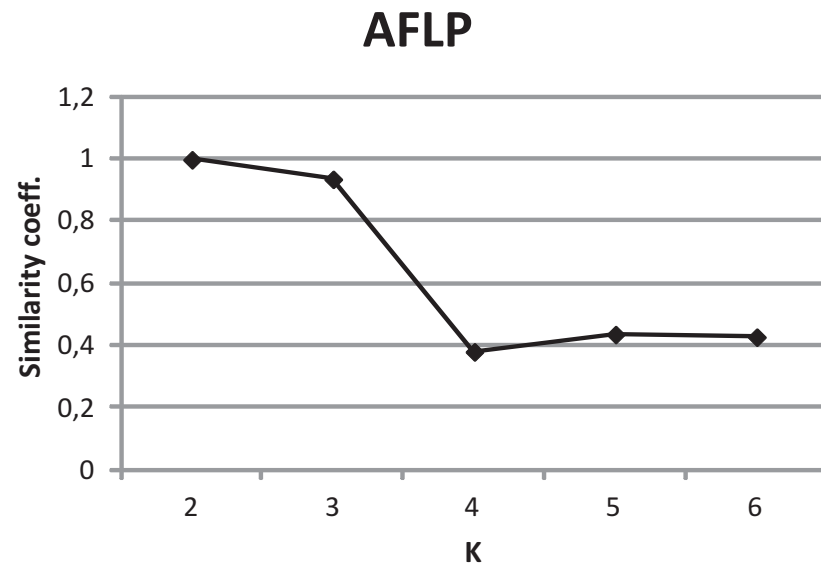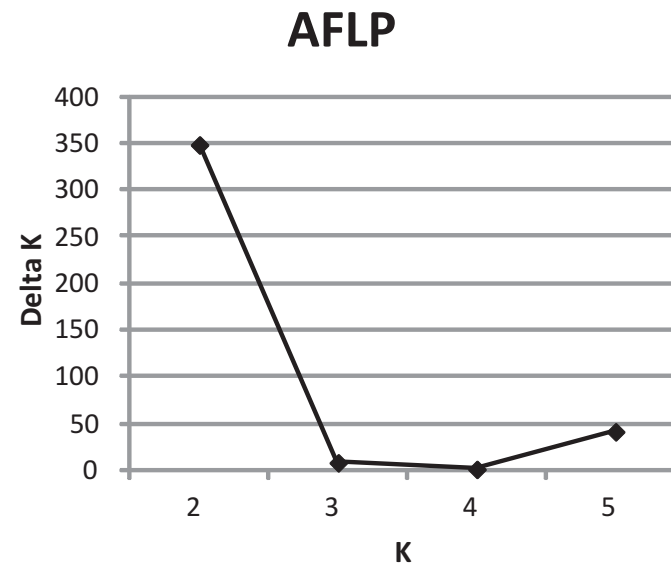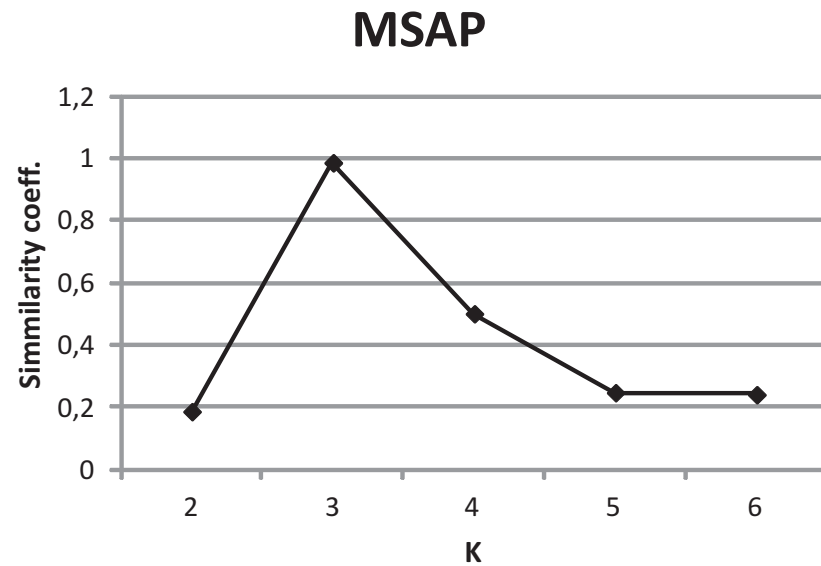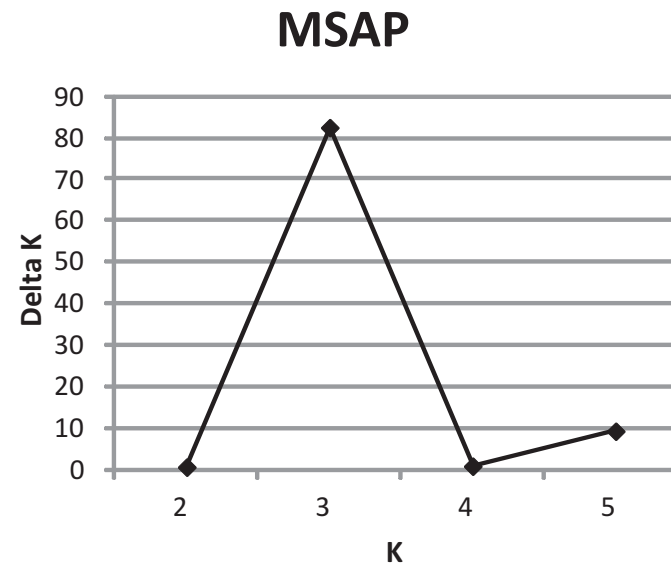

Supplement: Supplementary file 4 [file ece30003-3764-SD4.pdf]
