# Supplementary material for: Epigenetic patterns newly established after interspecific hybridization in natural populations of Solanum
Source: Ecol Evol. 2013 Sep 9;3(11):3764–79. doi: 10.1002/ece3.758 (PMC3810873; doi:10.1002/ece3.758)

Fig. S5 Scan to identify epiloci subject to selection using BayeScan. Each dot corresponds to an MSAP locus. F_{ST} is plotted against the $\log_{10}$ of the posterior odds (PO), which provides evidence whether the locus is subject to selection or not. Arrows on the x axis from left to right show the minimum threshold for strong, very strong, and decisive evidence for selection on Jeffreys' scale.

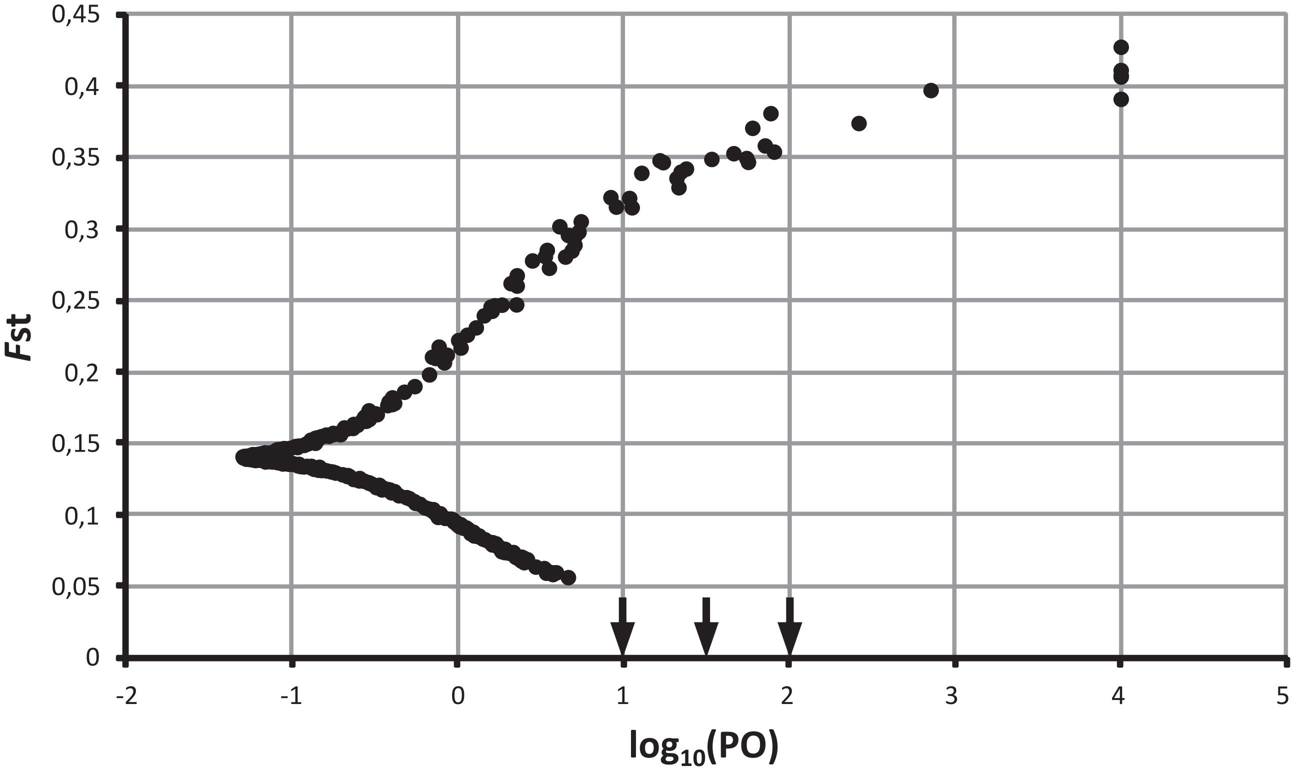

Supplement: Supplementary file 5 [file ece30003-3764-SD5.pdf]
